# An Overview About Figure-of-Eight Walk Test in Neurological Disorders: A Scoping Review

**DOI:** 10.3390/neurolint17070112

**Published:** 2025-07-21

**Authors:** Gabriele Triolo, Roberta Lombardo, Daniela Ivaldi, Angelo Quartarone, Viviana Lo Buono

**Affiliations:** IRCCS Centro Neurolesi Bonino Pulejo, 98124 Messina, Italy; gabriele.triolo@irccsme.it (G.T.); daniela.ivaldi@irccsme.it (D.I.); angelo.quartarone@irccsme.it (A.Q.); viviana.lobuono@irccsme.it (V.L.B.)

**Keywords:** F8WT, walking, assessment, stroke, Parkinson’s disease, multiple sclerosis

## Abstract

Introduction: The figure-of-eight walk test (F8WT) assesses gait on a curved path, reflecting everyday walking complexity. Despite recognized validity among elderly individuals, its application in neurological disorders remains inadequately explored. This scoping review summarizes evidence regarding F8WT use, validity, and clinical applicability among individuals with neurological disorders. Methods: A systematic literature search was conducted in the PubMed, Scopus, Embase, and Web of Science databases. After reading the full text of the selected studies and applying predefined inclusion criteria, seven studies, involving participants with multiple sclerosis (n = 3 studies), Parkinson’s disease (n = 2 studies), and stroke (n = 2 studies), were included based on pertinence and relevance to the topic. Results: F8WT demonstrated strong reliability and validity across various neurological populations and correlated significantly with established measures of gait, balance, and disease severity. Preliminary evidence supports its ability to discriminate individuals at increased fall risk and detect subtle motor performance changes. Discussion: The F8WT emerges as a valuable tool, capturing multifaceted gait impairments often missed by linear walking assessments. Sensitive to subtle functional changes, it is suitable for tracking disease progression and intervention efficacy. Conclusions: F8WT is reliable and clinically relevant, effectively identifying subtle, complex walking impairments in neurological disorders.

## 1. Introduction

Walking is a complex motor skill that entails dynamic integration between neural and bodily systems, required not only to execute gait but also to rapidly adapt to changes in environmental conditions and locomotor intentions [1,2,3]. Due to its complexity and its importance for the person, walking assessment is a key component in evaluating mobility, functional capacity, and rehabilitation progress in various populations, including those with neurological conditions, older adults, and individuals recovering from injury. Multiple clinical tests and technological tools are available to measure different aspects of walking ability, endurance, adaptability, and fatigability. Timed walk tests such as the Timed up and go (TUG), 2 min walk test (2MWT), 6 min walk test (6MWT), 10 m walking test (10MWT), and timed 25-foot walk (T25FW) measure walking speed, endurance, and overall walking capability [4,5,6,7]. These tests involve walking along a straight path, with few or no changes in direction, making them effective in standardized clinical settings but relatively less appropriate for evaluating the complex mobility demands of everyday life.

Notably, gait mechanics differ between straight and curved walking paths, particularly in terms of step characteristics and the positioning of body mass relative to the base of support. Straight-path walking involves a more symmetrical distribution of balance across the medial and lateral aspects of both feet [8,9]. By contrast, when navigating a curved path, individuals display reduced stride lengths on the inner leg compared to the outer leg, which covers a greater distance to complete the turn. This asymmetry is accompanied by a medial shift of the center of mass toward the inner foot and a prolonged stance phase on that side. During counterclockwise circular walking, the outer foot typically supports a medialized load, while clockwise walking results in a lateralized load distribution on the inner foot. These biomechanical adaptations are particularly relevant given that real-life ambulation often involves turning and curved trajectories. Turning imposes mechanical constraints inherent to curved-path progression, such as asymmetrical stride lengths, resultant torques, and the generation of centripetal forces to preserve dynamic equilibrium. Additionally, changes in head orientation during turning produce asymmetrical vestibular and cervical sensory inputs, which are likely reflected in the modulation of propulsive muscle patterns [10,11,12,13,14,15,16]. These observations highlight how walking in daily life is a highly complex motor activity that requires the coordinated activation of multiple brain regions, including those involved in balance control. One widely used clinical tool to assess balance is the Berg balance scale (BBS), a 14-item measure that quantitatively evaluates balance performance and fall risk in older community-dwelling adults through direct observation of functional tasks [17].

The figure-of-eight walk Test (F8WT), which has been validated primarily in older adult populations, involves curves in clockwise and counterclockwise directions, which is essential for real-world activity. The F8WT requires a person to walk a figure of 8 around two cones placed 5 feet apart. In its original form, it scores three components of skilled movement: time to completion, number of steps taken, and accuracy, i.e., the test must be completed within a 2-foot radius of the cones [18]. Unlike linear walking tests, such as the TUG, 2MWT, 6MWT, 10MWT, and T25FW, the F8WT requires continuous changes of direction and turning, which challenge anticipatory postural adjustments, spatial navigation, and motor planning. Curved-path walking has been shown to threaten dynamic stability and increase gait variability, revealing deficits not apparent in straight-line gait, especially in older adults and individuals with neurological or cognitive impairment [19].

Gait is frequently impaired in individuals with neurological disorders, making it essential to employ reliable and valid assessment tools capable of capturing the complexity of walking performance. This is particularly relevant in conditions such as multiple sclerosis (MS), Parkinson’s disease (PD), and stroke, where gait disturbances are highly prevalent and often evolve over time. Despite differences in underlying pathology, these disorders frequently lead to impaired gait and balance, which underscores the need for tools that can detect subtle deficits and monitoring functional mobility in clinical and research contexts [20].

Despite its relevance, to the best of our knowledge, the application of the F8WT in individuals with neurological disorders remains poorly explored. This gap underscores the need for a comprehensive synthesis of the current literature. Therefore, this scoping review aims to map the available evidence on the use, validity, and clinical applicability of the F8WT in neurological populations.

## 2. Materials and Methods

This scoping review research on “Pubmed”, “Scopus”, “Embase”, and “Web Of Science” was conducted from March to May 2025.

The PCC framework (Population, Concept, Context) [21] was utilized to establish well-defined and relevant objectives and eligibility criteria for this scoping review:

- P: People with neurological disorders;
- C: To explore the application of F8WT, its measurement properties, its reliability, and the possibility of distinguishing, through this test, subjects with a higher risk of falling;
- C: Studies conducted across various healthcare and rehabilitation settings, with no geographical or cultural restrictions.

The following string was used for databases:

- (“figure of eight walk” OR “figure-of-eight walk” OR “figure 8 walk” OR “figure eight walk” OR “figure of eight gait” OR “Figure-of-8 Walk Test”)

This review was conducted in accordance with the PRISMA-ScR checklist [22]. The protocol was registered on the Open Science Framework (OSF) with the following registration number: 10.17605/OSF.IO/QR758.

**Figure 1:**
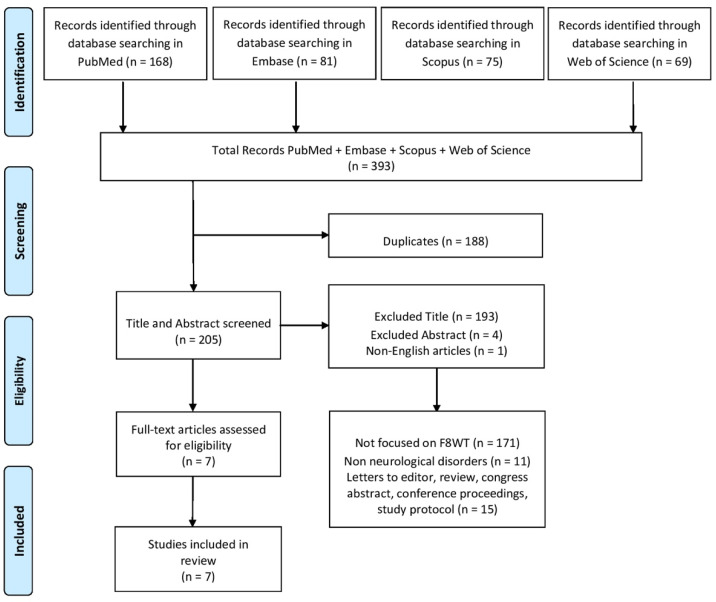
Study identification and screening process.

A PRISMA (Preferred Reporting Items for Systematic Reviews and Meta-Analyses) diagram was added to describe all steps of the search process (identification, screening, eligibility, and inclusion) for the collection and determination of qualified studies, as shown in Figure 1.

A total of 393 records were screened by three investigators (G.T., R.L., and D.I.) based on title, abstract, and full-text analysis. Any disagreements regarding study selection were first discussed among the reviewers and resolved by consensus; if necessary, a fourth investigator (V.L.B.) was consulted to make the final decision. Data extraction was then carried out independently by two reviewers (G.T. and R.L.) using a standardized form. Articles were included if they involved F8WT in people with neurological disease. Studies not written in English, due to risk of compromising methodological consistency, and studies involving animals, other assessments, orthopedic patients, or other types of interventions were excluded. Also, conference proceedings and systematic, narrative, and scoping reviews were excluded.

After deleting duplicates, 193 records were excluded by title screening. One record was excluded because it was not written in English. After abstract screening, four records were excluded because they were conference proceedings or poster abstracts. Following the full screening process, seven studies met the inclusion criteria and were included in the final analysis. Detailed characteristics of the selected studies are summarized in Table 1.

A formal critical appraisal of the methodological quality of the included studies was conducted using the Joanna Briggs Institute (JBI) critical appraisal checklist for Analytical Cross-Sectional Studies [23]. Eight criteria were assessed independently by two reviewers (G.T. and R.L.), with discrepancies resolved through discussion or with the involvement of a third reviewer (V.L.B.). The results of the quality appraisal are presented in Table 2.

**Table 1:** Main characteristics of the studies included.

Authors and Year	Population	Aim	Other Measures	Outcomes
Ozden F et al. (2022) [24]	52 RRMS	To assess the reliability and validity of F8WT and L Test.	L Test TUG T25FW EDSS	Reliable and valid test in mildly disabled PwMS. High correlation with TUG and T25FW, moderate correlation with EDSS.
Soke F et al. (2022) [25]	42 PwMS, 33 HSs	To evaluate the F8WT in PwMS for reliability, measurement error, validity, and fall risk discrimination.	TUG BBS ABC EDSS	The F8WT is a reliable tool for assessing walking and fall risk in PwMS. It can detect real changes in walking ability following interventions in ambulatory individuals with low disability, supporting its clinical applicability. Strong correlation with TUG and BBS.
Katirci Kirmaci Z et al. (2023) [26]	45 PwMS	To evaluate the reliability, validity, and MDC of the F8WT in mildly disabled PwMS.	BBS TUG T25FW FSST	The F8WT showed strong correlations with balance and mobility measures, supporting its clinical use to assess disease progression, treatment effects, and post-relapse impact in PwMS.
Lowry K et al. (2022) [27]	60 PwPD, 34 OA	To assess the validity of the F8WT in PwPD by examining its associations with gait, cognition, and physical function and its sensitivity to walking-skill deficits.	MOCA LLFDI DCI	The F8WT showed strong associations with gait speed, gait variability, cognition, and physical function in PwPD. The test differentiated physical function levels and effectively distinguished PwPD from healthy older adults, with higher sensitivity and specificity than straight-path gait speed.
Soke F et al. (2023) [28]	43 PwPD, 34 HSs	To assess the F8WT in PwPD by examining its reliability across different raters and over time as well as its validity through associations with related measures and its ability to distinguish between clinically relevant groups.	TUG 10MWT BBS ABC UPDRS H&Y	The F8WT showed excellent reliability and good concurrent validity in PwPD, with strong correlations with gait, balance, and disease severity measures. It effectively distinguished both PwPD from healthy individuals and fallers from non-fallers, supporting its clinical use for assessing walking ability.
Horata E et al. (2025) [29]	32 stroke patients	To assess the validity and reliability of the single- and dual-task F8WT among stroke patients.	SMMT NIHSS TUG 10MWT mFSST SSST	Single- and dual-task F8WT demonstrated good reliability and strong correlations with established mobility tests, supporting their validity.
Wong S et al. (2013) [30]	35 subjects with chronic stroke and 29 healthy elderly	To assess the F8WT’s reliability, its associations with stroke-specific impairments, and its ability to detect differences in advanced walking performance between stroke survivors and controls.	FMA-LE FTSTST Dynamometer 10MWT TUG BBS ABC	The F8WT showed excellent reliability and strong correlations with stroke-specific measures. It effectively distinguished stroke patients from healthy older adults, supporting its validity for assessing advanced walking ability in chronic stroke.

Legend: RRMS = relapsing—remitting multiple sclerosis, F8WT = figure-of-eight walk test, TUG = timed up and go, T25FW = timed 25-foot walk, pwMS = people with Multiple Sclerosis, HSs = healthy subjects, BBS = Berg balance scale, ABC = Activities-specific Balance Confidence Scale, FSST = four square step test, MDC = minimal detectable change, PwPD = people with Parkinson’s disease, OA = older adults, MOCA = Montreal Cognitive Assessment, LLFDI = Late-Life Function and Disability Instrument, DCI = Duke Comorbidity Index, 10MWT = 10 m walking test, UPDRS = Unified Parkinson’s Disease Rating Scale, H&Y = Hoehn and Yahr, SMMT = Standardized Mini Mental Test, NIHSS = National Institutes of Health Stroke Scale, mFSST = modified four square step test, SSST = Six-Spot Step Test, FMA-LE = Fugl–Meyer Motor Assessment for the lower extremities, FTSTST = Five times Sit to Stand Test.

**Table 2:** ICC and 95% confidence intervals across included studies.

Authors and Year	Neurological Population	ICC (Type)	Effect Size	Outcome	95%CI
Özden F et al. (2022) [24]	Multiple sclerosis	ICC (test–retest)	0.972	Completion time	0.97–0.99
Soke F et al. (2022) [25]	Multiple sclerosis	ICC (test–retest)	0.916	Completion time	0.85–0.95
Katirci Kirmaci Z et al. (2023) [26]	Multiple sclerosis	ICC (test–retest)	0.980	Completion time	0.96–0.98
Soke F et al. (2023) [28]	Parkinson’s disease	ICC (test–retest)	0.905	Completion time	0.82–0.94
Horata E et al. (2025) [29]	Stroke	ICC (test–retest)	0.938	Completion time	0.87–0.96
Wong S et al. (2013) [30]	Stroke	ICC (test–retest)	0.977	Completion time	0.95–0.98

Legend: ICC = intraclass correlation coefficient, CI = confidence interval.

## 3. Results

The studies included in the results were chosen following the selection process described in Section 2. Seven studies were deemed eligible for inclusion in this scoping review. The main characteristics of each included study are summarized in Table 1.

### 3.1. Multiple Sclerosis

The F8WT showed good inter- and intra-reliability, identifying comprehensive gait characteristics in mildly impaired people with MS. Preliminary evidence suggests that a completion time of less than 8.52 s could help distinguish individuals at greater risk of falling. The minimal detectable change (MDC) score, which is the smallest amount of change in the test score as distinct from changes due to random error or measurement variability, indicates that the test is able to detect small or subtle changes in motor performance related to walking and balance and is, therefore, particularly useful for identifying changes during periods of relapse or remission and monitoring treatment responses and disease progression.

### 3.2. Parkinson’s Disease

In the assessment of gait in people with Parkinson’s disease (PwPD), the F8WT demonstrated reliability and validity, supporting its usefulness in detecting walking impairments that may not be identified through standard tests. The MDC helped distinguish true differences between PwPD and healthy individuals, providing clinically meaningful information for evaluating the effectiveness of interventions [28]. Additionally, F8WT performance may be associated with an increased risk of falling [27,28].

### 3.3. Stroke

The F8WT is a reliable and valid assessment tool for walking in stroke patients. The MDC values detected through the administration of both the single- and dual-task F8WT were higher than those of PD and MS, indicating a higher risk of measurement error and variability. The test can be used to discriminate patients with chronic stroke from healthy elderly subjects [29,30].

### 3.4. Correlation with Other Motor Tests

A strong correlation with other motor tests was shown in the studies. In particular, a strong correlation was shown with other walking tests like the TUG [24,25,26,28,29,30], T25FW [24,26], and 10MWT [28,29,30]; balance tests like the BBS [25,26,28,30], four square step test [26], modified four square step test, and SSST [29]; and muscle strength tests like the FTSTST and FMA-LE [30].

### 3.5. Correlation with Disease Severity

A moderate correlation was shown with EDSS [24,25], UPDRS-total, UPDRS II, UPDRS III, and H&Y [28], suggesting that the test could serve as a predictor of disease severity.

### 3.6. Test–Retest Reliability

The test–retest reliability of the F8WT was assessed in six of the included studies, all of which reported intraclass correlation coefficients (ICCs) based on completion time. As summarized in Table 2, ICC values ranged from 0.905 to 0.980. Although these results consistently indicate excellent reliability, the studies were conducted in different neurological populations, preventing broad generalization of the results. The 95% confidence intervals were narrow in all cases, supporting the precision of these estimates.

**Figure 2:**
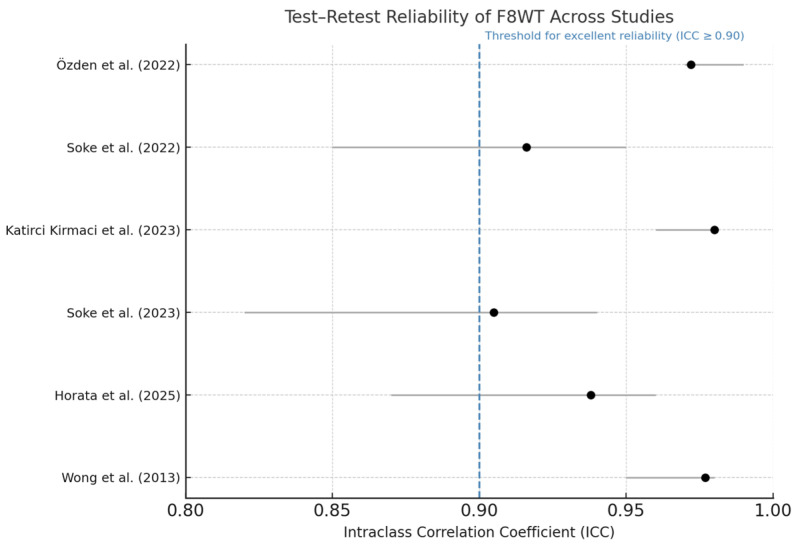
Forest plot of test–retest reliability for the F8WT. Dots indicate ICC; horizontal bars represent 95% confidence intervals. The dashed vertical line at ICC = 0.90 marks the conventional threshold for excellent reliability [24,25,26,28,29,30].

Table 2 presents test–retest ICC values for the F8WT across six studies involving different neurological populations, including individuals with MS, PD, and stroke. All reported ICCs were above 0.90, indicating excellent reliability of the completion time measure. Despite this consistency, the studies differ in several methodological aspects. Therefore, while the high ICCs suggest strong measurement stability, direct comparisons across populations are not methodologically appropriate. These findings support the test’s potential utility across various neurological conditions, but further population-specific validation is recommended. To enhance accessibility and support visual interpretation of the psychometric data, a forest plot summarizing the ICC values and their confidence intervals is provided in Figure 2.

### 3.7. Quality Appraisal

A methodological quality appraisal was conducted using the JBI critical appraisal checklist. Specifically, the appraisal involved eight criteria:

- Q1: “Clearly defined inclusion criteria”
- Q2: “Detailed description of study subjects and setting”
- Q3: “Valid and reliable measurement of the exposure”
- Q4: “Objective and standardized measurement of the condition”
- Q5: “Identification of confounding factors”
- Q6: “Strategies to deal with confounding factors”
- Q7: “Valid and reliable measurement of outcomes”
- Q8: “Appropriate statistical analysis”

The total represents the number of criteria that each study met (answered “Yes”) out of the eight possible criteria, expressed as a percentage. Higher percentages indicate better methodological quality and a lower risk of bias. A summary of the appraisal is presented in Table 3.

The results of the methodological appraisal indicate that all included studies clearly defined inclusion criteria, described participants and settings adequately, and employed valid and reliable methods to measure outcomes. However, criteria related to identifying and managing confounding factors (Q5 and Q6) were occasionally unclear, suggesting an area for methodological improvement in future studies. Overall, the included studies demonstrated a satisfactory methodological quality, supporting the robustness of the synthesized findings despite these limitations.

## 4. Discussion

This scoping review examines the application of the F8WT in people with neurological disorders, a potentially important tool that can be used to record parameters similar to those needed in everyday life, such as walking on a curved path. Compared to widely used gait assessments such as the TUG, 10MWT, and T25FW, which primarily evaluate linear walking and basic turning, the F8WT introduces continuous directional changes requiring greater dynamic balance, spatial planning, and gait adaptability. These features make the F8WT more ecologically valid, as curved-path walking is more representative of real-world ambulation. Moreover, the F8WT may detect subtle impairments in motor control or cognitive–motor interaction that straight-line tests may miss, particularly in populations with early neurological changes. Therefore, the F8WT should not be considered a substitute but rather a complementary assessment to standard gait measures, potentially enhancing the sensitivity of clinical evaluations. Across the reviewed studies, the F8WT consistently emerged as an objective and responsive tool for assessing curved-path walking in different neurological conditions. Its capacity to detect even subtle functional changes reinforces its potential for use in tracking disease progression and evaluating intervention outcomes. Unlike linear walking tests, the F8WT challenges postural control and directional transitions, offering clinically relevant insights into mobility that may otherwise go undetected in standard gait assessments [24,25,26,27,28,29,30].

Other authors investigated the use of the F8WT in various populations, including older adults, demonstrating its validity not only in a clinical setting but also in home environments. Compared to other timed gait assessments based on straight-path walking, curved-path walking could provide parameters that are more representative of functional conditions in everyday life [31].

Furthermore, given its ability to quantify both completion time and step count, the F8WT may serve as a useful tool for identifying individuals at increased risk of falls. Therefore, the exploration of meaningful cut-off values could further support its application in clinical decision-making and optimize sensitivity and specificity. A completion time of ≤9.09 s distinguished individuals with excellent self-reported global mobility, while a threshold of ≤9.27 s identified those with excellent or very good mobility. Additionally, taking ≤17 steps was associated with excellent to good global balance. Notably, individuals performing above these thresholds had a significantly lower incidence of adverse outcomes, including falls, emergency department visits, and hospitalizations, with relative reductions ranging from 44% to 59% [32]. Interestingly, when performed with an added motor task, the F8WT has shown greater sensitivity in identifying fall risk compared to its standard version or when performed with a cognitive task in older adults. This suggests that incorporating a concurrent motor challenge may enhance the discriminatory power of the test, offering clinicians a valuable approach for the early identification of older adults at higher risk of falling [33]. Accordingly, the inclusion of additional test conditions may provide clinicians with a more comprehensive understanding of patients’ functional abilities, offering insights not only limited to the clinical setting but also reflective of daily life demands. As reported in another study, walking while carrying a load or on uneven terrain has further reinforced the psychometric robustness of the F8WT, even in individuals with lower limb amputation. Time to complete F8WT and number of steps taken showed moderate to good correlations with performance-based mobility measures and fair to good correlations with self-reported measures of mobility. These findings highlight the potential of condition-specific modifications not only to improve measurement precision but also to tailor assessment protocols to the functional profiles of different clinical populations [34].

Recent advances in digital health have enabled automated versions of traditional mobility assessments. The automated F8WT (aF8WT) demonstrated excellent agreement with the manual version and strong test–retest reliability, confirming its suitability for clinical and remote applications. Beyond reducing inter-rater variability, the automated system provides real-time data analysis and minimizes the need for operator expertise, supporting its integration into routine functional monitoring [35].

The F8WT may also serve as a valuable tool to assess the effectiveness of interventions aimed at improving lower limb strength. A significant correlation between F8WT completion time and hip external rotator strength has been observed in healthy young males [36]. This finding may have particular relevance for clinical populations with neurological disorders, where proximal muscle weakness and impaired motor control often compromise turning ability and dynamic balance. In this context, the F8WT could be used to monitor functional improvements resulting from targeted rehabilitation strategies.

The F8WT involves not only postural and gait control but also executive functions—including planning, cognitive flexibility, and spatial navigation—making it a cognitively demanding task, especially compared to straight-path walking [37,38]. There is a modest correlation between total Montreal Cognitive Assessment (MOCA) score and F8WT performance in pwPD [27]. The MOCA is a cognitive screening tool, a composite measure of cognition across several domains including executive function, short-term memory, and language. Even in older adults, there is a modest correlation between F8WT and Trail Making B, a widely used measure of cognitive flexibility and divided attention, both in relation to number of steps and time to complete the test [18]. Studying the relationship between cognitive tests and motor skills such as walking may serve as an early indicator of cognitive decline. Research exploring how conditions like Mild Cognitive Impairment (MCI) affect walking ability is increasing. Older adults with MCI consistently show slower walking speeds, reduced mobility, and poorer performance on complex walking tasks compared to cognitively healthy peers. MCI is linked to reduced gait smoothness, especially during tasks requiring high control, such as walking with head turns, indicating subtle motor control deficits [39,40,41].

### 4.1. Implications for Practice and Future Studies

To improve reproducibility and comparability, standardization of the F8WT administration protocol is recommended. Cone spacing should be set at 1.52 m (5 feet); participants should walk at a comfortable, self-selected pace while wearing regular footwear; and trials should include one familiarization trial followed by two timed attempts. Measured outcomes should not only include completion time but also systematically investigate step count and gait smoothness, in line with the parameters outlined in the original F8WT [18], to enable clinicians to identify nuanced gait alterations that are not detectable through completion time alone. In addition, the clinical value of the F8WT may be further enhanced when integrated into multimodal assessment or rehabilitation protocols. Combining functional gait tests with neuromodulatory or sensorimotor interventions has been shown to improve diagnostic sensitivity and therapeutic outcomes in other populations [42,43]. In this context, the F8WT could serve not only as a monitoring tool but also as a dynamic task to reinforce motor adaptation and balance training. Future research should explore the synergistic effects of combining curved-path walking assessments with targeted interventions in neurological rehabilitation. Future directions should further explore the use of the F8WT in neurological populations, particularly in individuals with dementia, where early motor–cognitive signs may support timely diagnosis and intervention. The integration of digital technologies for automated test administration could enhance the objectivity, scalability, and clinical feasibility of the F8WT. Recent technological advances have enabled aF8WT using digital systems. A digital version of the F8WT has recently been validated, demonstrating excellent agreement and test–retest reliability when compared to manual assessment, suggesting its feasibility for clinical implementation [35]. Moreover, motion-capture systems, inertial sensors, and LiDAR-based devices have been tested to enhance gait analysis, offering high-resolution spatial and temporal data while preserving ecological validity. Integrating such technologies with the F8WT could improve measurement precision, enable remote monitoring, and facilitate scalable implementation in both clinical and home-based settings. Moreover, the test may hold promise as a potential biomarker for the early detection of neurodegenerative disorders. Correlations with fall risk, using objective and sensor-based measures, should also be a priority for future research to support the F8WT’s role in preventive clinical strategies. Future studies might also research the correlation between cognitive and motor functions; in this sense, gait analysis provided by the F8WT could be a sensitive marker for neurological populations.

### 4.2. Limitations

This study has some limitations. The original F8WT records not only the time required to complete the test but also the number of steps and the smoothness of gait throughout the task. However, the majority of the studies included in this review assessed the time required to complete the test, with limited consideration given to parameters such as the number of steps taken or the fluency of gait. Notably, only one study investigated the relationship between the number of steps needed to complete the test and a validated instrument measuring participants’ functional abilities and limitations in performing activities of daily living [27]. Moreover, only seven studies met the inclusion criteria, limiting the breadth of available evidence. The heterogeneity of outcome measures and test protocols across studies also hinders direct comparison and limits the ability to draw generalizable conclusions. Future studies should define a unique protocol for the administration of the test and for the parameters to be analyzed. Furthermore, with the advancement of artificial intelligence and machine learning techniques, future studies should explore the role of proprioception and its influence on gait asymmetry and turning performance, particularly in the context of rehabilitation [44,45,46,47].

## 5. Conclusions

In conclusion, the F8WT has proven to be a reliable and valid tool for assessing walking ability in patients with neurological disorders. It is a reliable tool that captures complex gait features often missed by linear walking tests and shows strong correlations with established motor assessments such as the TUG, T25FW, 10MWT, and BBS. Future studies should aim to strengthen these correlations by further investigating neurological populations. Another important area to explore involves the relationship between F8WT performance and patients’ cognitive abilities, such as attention, memory, and visuospatial skills, which remain understudied. Furthermore, exploring the potential offered by technology, including the digitalization of the test, may open new avenues for its use in both clinical and research settings.

## Figures and Tables

**Table 3:** Methodological quality appraisal of included studies.

Authors and Year	Q1	Q2	Q3	Q4	Q5	Q6	Q7	Q8
Özden F et al. (2022) [24]	Y	Y	Y	Y	Y	Y	Y	Y
Soke F et al. (2022) [25]	Y	Y	Y	Y	U	N	Y	Y
Katirci Kirmaci Z et al. (2023) [26]	Y	Y	Y	Y	U	U	Y	Y
Lowry K et al. (2022) [27]	Y	Y	Y	Y	Y	Y	Y	Y
Soke F et al. (2023) [28]	Y	Y	Y	Y	U	U	Y	Y
Horata E et al. (2025) [29]	Y	Y	Y	Y	Y	U	Y	Y
Wong S et al. (2013) [30]	Y	Y	Y	Y	Y	Y	Y	Y
Total %	100.0	100.0	100.0	100.0	57.14	42.85	100.0	100.0

Legend: Y = Yes, N = No, U = unclear.

## Data Availability

Not applicable.

## Abbreviations

The following abbreviations are used in this manuscript:

F8WT	Figure-of-eight walk test
TUG	Timed up and go
2MWT	2 min walk test
6MWT	6 min walk test
10MWT	10 m walking test
T25FW	Timed 25-foot walk
BBS	Berg balance scale
RRMS	Relapsing–remitting multiple sclerosis
pwMS	People with multiple sclerosis
HS	Healthy subject
ABC	Activities-specific Balance Confidence Scale
FSST	Four square step test
MDC	Minimal detectable change
PwPD	People with Parkinson’s disease
OA	Older adults
MOCA	Montreal Cognitive Assessment
LLFDI	Late-Life Function and Disability Instrument
DCI	Duke Comorbidity Index
UPDRS	Unified Parkinson’s Disease Rating Scale
H&Y	Hoehn and Yahr
SMMT	Standardized Mini Mental Test
NIHSS	National Institutes of Health Stroke Scale
mFSST	Modified four square step test
SSST	Six-Spot Step Test
FMA-LE	Fugl–Meyer Motor Assessment for the lower extremities
FTSTST	Five times Sit to Stand Test
